# Oxymatrine Inhibits Epithelial‐Mesenchymal Transition to Alleviate Airway Remodeling in Chronic Obstructive Pulmonary Disease by Suppressing the TGF‐β1/Smad Pathway

**DOI:** 10.1002/kjm2.70118

**Published:** 2025-10-28

**Authors:** Shuang Zhou, Ju‐Xiang Zhu, Jing Li

**Affiliations:** ^1^ Department of Pulmonary Diseases Xiangyang Traditional Chinese Medicine Hospital (Xiangyang Institute of Traditional Chinese Medicine) Xiangyang Hubei China; ^2^ Department of General Medicine Zhongxiang People's Hospital Zhongxiang Hubei China

**Keywords:** chronic obstructive pulmonary disease, epithelial‐mesenchymal transition, inflammation, oxymatrine, TGF‐β1/Smad

## Abstract

This study explored the therapeutic efficacy of oxymatrine (OMT, C_15_H_24_N_2_O_2_) in a murine model of cigarette smoke (CS)‐induced chronic obstructive pulmonary disease (COPD) and elucidated its underlying mechanisms. A COPD model was established in mice through prolonged exposure to CS, followed by intraperitoneal administration of OMT (50 mg/kg) over 3 months. Lung inflammation and fibrosis were evaluated using histopathological techniques (hematoxylin–eosin and Masson staining), enzyme‐linked immunosorbent assays (ELISA), and pulmonary function tests. The expression of epithelial‐mesenchymal transition (EMT) markers and components of the transforming growth factor‐β1 (TGF‐β1)/Smad signaling pathway were assessed via Western blot and immunofluorescence. In vitro, human bronchial epithelial (HBE) cells were exposed to CS extract (CSE, 10% v/v) or TGF‐β1 (10 ng/mL), with or without treatment using OMT (80 μM) or the TGF‐β receptor I inhibitor SB525334 (10 μM). Cell viability, EMT marker expression, and Smad2/3 phosphorylation were measured. In vivo, OMT significantly reduced CS‐induced airway inflammation, as evidenced by decreased inflammatory cell infiltration and lower proinflammatory cytokines in bronchoalveolar lavage fluid. OMT treatment improved lung function by restoring compliance, reducing airway resistance, and enhancing forced expiratory capacity. Similarly, OMT alleviated CS‐induced pulmonary fibrosis and EMT, as shown by decreased collagen deposition and normalization of EMT marker expression (E‐cadherin, vimentin, and α‐smooth muscle actin [α‐SMA]) in lung tissues. In HBE cells, OMT counteracted EMT induced by CSE or TGF‐β1, significantly suppressing Smad2/3 phosphorylation and nuclear translocation. Mechanistic analysis revealed that OMT inhibited activation of the TGF‐β1/Smad signaling pathway in both in vivo and in vitro models. Moreover, combining OMT and SB525334 increased EMT suppression in CSE‐exposed HBE cells. Importantly, safety evaluations showed no histopathological abnormalities in major organs following OMT administration. In conclusion, these findings indicate that OMT mitigates COPD progression by targeting the TGF‐β1/Smad pathway, supporting its potential as a safe and effective therapeutic option for COPD management.

## 1 Introduction

Chronic obstructive pulmonary disease (COPD) is a serious respiratory condition and is expected to rank as the third leading cause of death worldwide by 2030 [1]. This progressive disease is characterized by sustained airflow obstruction and a gradual decline in lung function, largely resulting from pathological airway remodeling and chronic inflammation triggered by exposure to inhaled toxic substances [2]. Tobacco smoking remains the predominant risk factor, accounting for 90% of COPD‐related mortality [3], with cigarette smoke (CS) inducing oxidative stress and sustained inflammation as key drivers of pathogenesis [4]. Although early‐stage COPD is manageable through prevention and symptom control [5], current pharmacological strategies, primarily corticosteroids for inflammation suppression and bronchodilators for airway dilation [6], fail to halt disease progression. Current treatments mainly reduce the frequency of exacerbations but fail to address the core pathophysiological processes of the disease. This limitation highlights a critical need for new therapeutic strategies that directly target airway remodeling and inflammatory pathways to more effectively modify the course of COPD.

Airway remodeling, a hallmark of COPD‐related pulmonary dysfunction, results from extracellular matrix imbalance driving tissue fibrosis [7]. Prolonged exposure to harmful stimuli induces epithelial‐mesenchymal transition (EMT), a cellular reprogramming process in which polarized epithelial cells transform into mesenchymal‐like cells, gaining increased migratory ability, resistance to apoptosis, and matrix‐degrading functions [8]. Although EMT plays a vital role in normal physiological processes such as embryonic development, its aberrant activation contributes to persistent airway remodeling in COPD [9]. Emerging evidence positions EMT inhibition as a promising therapeutic strategy, though optimal molecular targets require further exploration [10]. At the core of this process lies the transforming growth factor‐β1 (TGF‐β1)/Smad signaling pathway, in which CS‐induced overexpression of TGF‐β1 [11] triggers Smad2/3 phosphorylation [12], therefore promoting collagen accumulation [13] and worsening clinical symptoms [14]. Targeted suppression of this pathway demonstrates therapeutic potential by mitigating EMT‐driven airway remodeling.

Emerging evidence highlights natural compounds as promising COPD therapeutics, targeting fibrosis, oxidative stress, and inflammation [15]. Oxymatrine (OMT, C_15_H_24_N_2_O_2_; molecular weight: 264.36 g/mol), a bioactive alkaloid derived from *Sophora flavescens* , exhibits a wide range of pharmacological properties, including anti‐fibrotic, anti‐inflammatory, and antioxidant effects [16, 17]. Preclinical studies reveal its efficacy in lung injury models: OMT attenuates ischemia–reperfusion injury by modulating cytokine balance and suppressing apoptosis [18], alleviates lipopolysaccharide‐induced acute lung injury by activating the epithelial sodium channel [19], and mitigates pulmonary fibrosis by blocking TGF‐β1/Smad signaling [20]. Although OMT's modulation of the TGF‐β1 pathway has demonstrated anti‐fibrotic effects in various disease models [21, 22, 23], its potential therapeutic role in CS‐induced COPD, especially via the regulation of EMT, has yet to be investigated.

This study is the first to explore the therapeutic potential of OMT in COPD pathogenesis using complementary in vitro models of human bronchial epithelial cells stimulated with CS extract (CSE) and in vivo models involving CS‐exposed mice. It is proposed that OMT inhibits EMT‐driven airway remodeling in COPD by targeting the TGF‐β1/Smad signaling pathway, representing a novel therapeutic approach not previously reported for this alkaloid in the context of COPD.

## 2 Materials and Methods

### 2.1 Preparation of CSE

CSE was prepared following established protocols [24] with modifications. Briefly, 3R4F reference cigarettes (University of Kentucky, Lexington, KY, USA; 0.76 mg nicotine and 9.4 mg tar per cigarette) were combusted, and smoke was collected using a 50‐mL glass syringe (Hamilton Company, Reno, NV, USA) at a standardized rate of 5 min per cigarette. CS was bubbled through 10 mL serum‐free Dulbecco's Modified Eagle Medium (DMEM; Gibco, Waltham, MA, USA). The resulting suspension was adjusted to a pH of 7.4 using 1 M NaOH (Sigma‐Aldrich, St. Louis, MO, USA) and then sterilized by filtration through a 0.22‐μm polyethersulfone membrane (Merck Millipore, Burlington, MA, USA). This stock solution, 100% CSE, was freshly diluted with DMEM to the desired working concentrations immediately before use.

### 2.2 COPD Mouse Model

Eight‐week‐old male C57BL/6J mice (18–20 g) were sourced from Charles River Laboratories (Beijing, China) and maintained under controlled environmental conditions: a temperature of 23°C ± 3°C, humidity at 50% ± 10%, and a 12‐h light/dark cycle. Animals were provided with unrestricted access to food and water. All experimental protocols were approved by the Laboratory Animal Welfare & Ethics Committee of Bestcell Model Biological Center (Approval No. BSMS 2023–05‐26A; Date: May 23, 2023). Following a 7‐day acclimatization, COPD was induced as described [25] using 3R4F reference cigarettes. Mice underwent whole‐body smoke exposure in a Plexiglas inhalation chamber (EMMS, Hampshire, UK): 9 cigarettes/h, 4 h/day, 6 days/week for 12 weeks. Sham controls received filtered air exposure under identical conditions. Mice were randomized into four groups (*n* = 8/group): Sham, Air exposure + intraperitoneal saline (0.9% NaCl; Sigma‐Aldrich, St. Louis, MO, USA); Sham + OMT (C_15_H_24_N_2_O_2_; molecular weight: 264.36 g/mol), Air exposure + OMT (50 mg/kg; Rayzebio, Cat. no. LZR5548, Shanghai, China); COPD, CS exposure + saline; COPD + OMT, CS exposure + OMT. OMT (HPLC purity ≥ 98%) or vehicle was administered 1 h pre‐exposure daily. The dosage was selected based on previous studies [26].

### 2.3 Sample Collection Procedures

Mice were anesthetized by intraperitoneal injection of pentobarbital sodium (50 mg/kg; Sigma‐Aldrich, St. Louis, MO, USA). After dissecting the cervical skin and muscles, tracheal intubation was performed using a 22G polyethylene catheter (BD Biosciences, Franklin Lakes, NJ, USA). Bronchoalveolar lavage fluid (BALF) was collected through three consecutive installations and aspirations of 0.6 mL ice‐cold sterile PBS (Sigma‐Aldrich, St. Louis, MO, USA), achieving a recovery rate exceeding 85%. The BALF was centrifuged at 1000 × *g* for 5 min at 4°C to separate the supernatant. Blood samples were drawn via puncture of the abdominal aorta using heparinized syringes (Terumo Medical Corporation, Tokyo, Japan), and serum was isolated by centrifugation at 3000 × *g* for 15 min at 4°C. For tissue processing, the left lungs were fixed in 10% neutral‐buffered formalin (Thermo Fisher Scientific, Waltham, MA, USA) for 48 h at 4°C for histopathological examination. The right lungs were rapidly snap‐frozen in liquid nitrogen (Airgas, Radnor Township, PA, USA) and stored at −80°C in a Revco ULT freezer (Thermo Fisher Scientific, Waltham, MA, USA) until protein extraction and immunoblotting analyses were performed.

### 2.4 Lung Function Assessment

After anesthesia with pentobarbital sodium, mice were positioned supine and underwent tracheotomy below the larynx using an 18G polyethylene cannula (Data Sciences International, New Brighton, MN, USA). Pulmonary function was assessed using the DSI Buxco FinePointe PFT System (Data Sciences International, Wilmington, NC, USA) following the manufacturer's instructions. Measurements included functional residual capacity, total lung capacity, lung resistance, chord compliance, forced vital capacity (FVC), and the ratio of forced expiratory volume at 50 ms to FVC (FEV50/FVC). The respiratory rate was maintained at 150 breaths per minute throughout the procedure using mechanical ventilation (Harvard Apparatus, Holliston, MA, USA).

### 2.5 Histological Examination

Left lung tissues fixed in 10% neutral‐buffered formalin underwent standard processing, including dehydration in graded ethanol series (Sigma‐Aldrich, St. Louis, MO, USA), xylene clearing (Merck KGaA, Darmstadt, Germany), and paraffin embedding (Paraplast; McCormick Scientific, St. Louis, MO, USA). Tissue sections (5 μm) were cut using a Leica RM2235 microtome (Leica Biosystems, Wetzlar, Germany), deparaffinized, and stained with hematoxylin–eosin (H&E; Yeason Biotech, Cat. no. 60524ES60, Shanghai, China) and Masson (Yeason Biotech, Cat. no. 60532ES58, Shanghai, China) kits. Histopathological evaluation was performed using a Nikon Eclipse Ci‐L brightfield microscope (Nikon Corporation, Tokyo, Japan), with inflammatory cell infiltration severity quantified via a semi‐quantitative scoring system (0–4 scale: 0 = absent, 4 = severe).

### 2.6 Immunofluorescence Analysis

Paraffin‐embedded lung sections (5 μm thick) were subjected to antigen retrieval in citrate buffer (pH 6.0; Sigma‐Aldrich, St. Louis, MO, USA) at 95°C for 20 min, followed by blocking with 5% donkey serum (Abcam, Cambridge, UK) for 1 h at room temperature. The sections were then incubated overnight at 4°C with primary antibodies: rabbit anti‐E‐Cadherin (1:200; Affinity Biosciences, Cat. no. AF0131, Cincinnati, OH, USA) and rabbit anti‐α‐smooth muscle actin (α‐SMA) (1:200; Affinity Biosciences, Cat. no. AF1032, Cincinnati, OH, USA). This was followed by a 1‐h incubation with Alexa Fluor 594‐conjugated donkey anti‐rabbit IgG (1:500; AmyJet Scientific, Cat. no. 711‐585‐152, Wuhan, China). Nuclei were counterstained with 4′,6‐diamidino‐2‐phenylindole (DAPI, 1 μg/mL; Sigma‐Aldrich, St. Louis, MO, USA) for 5 min. Fluorescent images were acquired using a Nikon A1R confocal microscope (Nikon Corporation, Tokyo, Japan). The fluorescence intensity of E‐Cadherin and α‐SMA was quantified as relative fluorescence units (RFU) using NIS‐Elements AR 5.21 software (Nikon Corporation, Tokyo, Japan).

### 2.7 Cell Culture and Treatment

Human bronchial epithelial (HBE) cells (Boke Biotechnology, Shanghai, China) were cultured in RPMI‐1640 medium (Gibco, Waltham, MA, USA) supplemented with 10% fetal bovine serum (Gibco, Waltham, MA, USA) at 37°C in a 5% CO_2_ atmosphere. Cell viability was evaluated using the Cell Counting Kit‐8 (CCK‐8; Absin Bioscience, Cat. no. abs50003, Shanghai, China). Cells were seeded at a density of 1 × 10^4^ cells per well in 96‐well plates (Corning, Corning, NY, USA) and serum‐starved for 24 h before treatment with varying concentrations of cigarette smoke extract (CSE; 0%–20%) or OMT (1–200 μM) in serum‐free medium. Following treatment, 10 μL CCK‐8 reagent was added to each well and incubated for 2 h. Absorbance was measured at 450 nm using a Synergy H1 microplate reader (BioTek Instruments, Winooski, VT, USA). To induce EMT, confluent HBE cells were treated with 10% CSE or TGF‐β1 (10 ng/mL; PeproTech, Cat. no. PT‐100‐21C, Cranbury, NJ, USA) [27]. To evaluate OMT's effects, cells were pretreated with 80 μM OMT (24 h) before 48 h of CSE/TGF‐β1 stimulation. Post‐treatment, cells were harvested for downstream analyses.

### 2.8 Enzyme‐Linked Immunosorbent Assay (ELISA)

Cytokine levels including Interleukin‐6 (IL‐6; Mouse IL‐6 ELISA Kit, Cat. no. M6000B, R&D Systems, Minneapolis, MN, USA), Tumor Necrosis Factor‐α (TNF‐α; Mouse TNF‐α ELISA Kit, Cat. no. 88‐7324‐22, Thermo Fisher Scientific, Waltham, MA, USA), Interleukin‐1β (IL‐1β; Mouse IL‐1β ELISA Kit, Cat. no. ab197742, Abcam, Cambridge, UK), and Transforming Growth Factor‐β1 (TGF‐β1; Mouse TGF‐β1 Quantikine ELISA Kit, Cat. no. MB100B, R&D Systems, Minneapolis, MN, USA; Human TGF‐β1 ELISA Kit, Cat. no. PT880, Beyotime, Shanghai, China) were measured in bronchoalveolar lavage fluid (BALF), serum, and HBE cell culture supernatants according to the manufacturer's instructions. Absorbance readings were taken at 450 nm using a Bio‐Rad iMark Microplate Reader (Bio‐Rad Laboratories, Hercules, CA, USA), with a wavelength correction set at 570 nm. All assays were performed in triplicate, using freshly prepared dilutions of recombinant standards supplied with each kit.

### 2.9 Western Blot Analysis

Total protein was isolated from lung tissues and HBE cells using RIPA lysis buffer (Solarbio Life Sciences, Beijing, China). Protein concentrations were determined by using a BCA assay (Solarbio Life Sciences, Beijing, China). Equal amounts of protein (30 μg per lane) were resolved on 10% SDS‐PAGE gels (Bio‐Rad Laboratories, Hercules, CA, USA) and subsequently transferred onto PVDF membranes (Merck Millipore, Burlington, MA, USA). Membranes were blocked with 5% non‐fat milk (Beyotime, Shanghai, China) for 1 h at room temperature, then incubated overnight at 4°C with primary antibodies from Affinity Biosciences (Cincinnati, OH, USA): Vimentin (1:1000; Cat. no. AF7013), E‐Cadherin (1:1000; Cat. no. AF0131), α‐SMA (1:1000; Cat. no. AF1032), TGF‐β1 (1:1000; Cat. no. AF1027), TGF‐β receptor I TGF‐βR1 (1:1000; Cat. no. AF5347), p‐Smad2 (1:1000; Cat. no. AF3449), Smad2 (1:1000; Cat. no. AF6449), p‐Smad3 (1:1000; Cat. no. AF3362), Smad3 (1:1000; Cat. no. AF6362), and GAPDH (1:3000; Cat. no. AF7021). After washes, membranes were incubated for 2 h with HRP‐conjugated goat anti‐rabbit IgG (1:3000; Affinity Biosciences, Cat. no. S0001, Cincinnati, OH, USA) at room temperature. Protein bands were visualized using the SuperSignal West Pico PLUS Chemiluminescent Substrate (Thermo Fisher Scientific, Waltham, MA, USA) on an Amersham Imager 600 (GE Healthcare, Chicago, IL, USA), with densitometric analysis performed in ImageJ 1.53 k (National Institutes of Health, Bethesda, MD, USA).

### 2.10 Immunofluorescence Detection of Smad2/3 Nuclear Translocation

HBE cells were pretreated with 80 μM OMT for 24 h, followed by exposure to 10% CSE for 30 min. Cells were then fixed with 4% paraformaldehyde (Sigma‐Aldrich, St. Louis, MO, USA) for 15 min, permeabilized using 0.2% Triton X‐100 (Sigma‐Aldrich, St. Louis, MO, USA) for 10 min, and blocked with 5% donkey serum for 1 h at room temperature. Subsequently, cells were incubated overnight at 4°C with primary antibodies against Smad2 (rabbit anti‐Smad2, 1:200; Affinity Biosciences, Cat. no. AF6449, Cincinnati, OH, USA) and Smad3 (rabbit anti‐Smad3, 1:200; Affinity Biosciences, Cat. no. AF6362, Cincinnati, OH, USA). After washing, cells were stained with Alexa Fluor 594‐conjugated donkey anti‐rabbit IgG (1:500; AmyJet Scientific, Cat. no. 711‐585‐152, Wuhan, China) for 1 h at 37°C. Nuclei were counterstained with DAPI (1 μg/mL) for 5 min, and images were captured using a Nikon A1R confocal microscope. Nuclear Smad2/3 fluorescence intensity was quantified as RFU using NIS‐Elements AR 5.21 software.

### 2.11 Statistical Analysis

Data analysis was conducted using GraphPad Prism 9.0 (GraphPad Software, San Diego, CA, USA), with results presented as mean ± standard deviation (SD). Differences between groups were evaluated by one‐way analysis of variance followed by Tukey's post hoc test. Each experiment included at least three independent biological replicates. Statistical significance was set at *p* < 0.05.

## 3 Results

### 3.1 OMT Treatment Alleviates CS‐Induced Airway Inflammation and Pulmonary Dysfunction in COPD Mice

**FIGURE 1:**
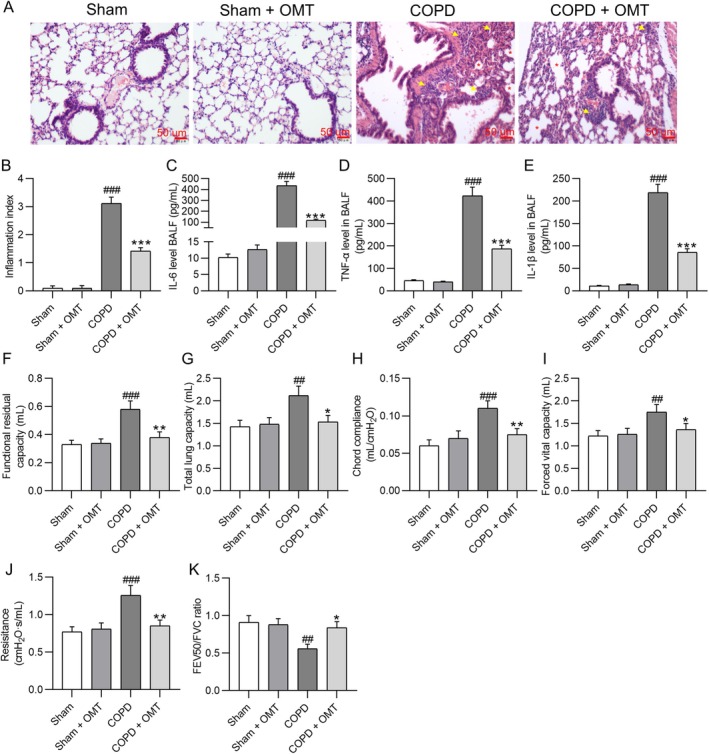
OMT treatment alleviates CS‐induced airway inflammation and pulmonary dysfunction in COPD mice. (A, B) Representative images of H&E‐stained lung tissue sections (scale bar: 50 μm) with quantitative assessment of airway inflammation. Yellow arrows indicate inflammatory cell infiltration in peribronchial and perivascular areas; red asterisks mark alveolar wall damage and emphysema‐like alveolar enlargement. (C, E) ELISA measurements of BALF levels of proinflammatory cytokines (IL‐6, TNF‐α, IL‐1β). Data are presented as mean ± SD in pg/mL. (F–K) Pulmonary function parameters: Functional residual capacity (mL), total lung capacity (mL), chord compliance (mL/cmH_2_O), forced vital capacity (mL), lung resistance (cmH_2_O·s/mL), and forced expiratory volume at 50 ms (FEV50/FVC). *N* = 8 mice/group. ^##^ *p* < 0.01, ^###^ *p* < 0.001 versus sham; *p < 0.05, **p < 0.01, ***p < 0.001 versus COPD.

After euthanasia, lung tissues from mice were stained with H&E, revealing pronounced inflammatory cell infiltration in CS‐exposed COPD mice (inflammatory score: 3.12 ± 0.22) compared to sham controls (0.10 ± 0.08, *p* < 0.001), along with evident alveolar wall damage. Treatment with OMT (50 mg/kg) significantly alleviated both alveolar destruction and inflammation, reducing the inflammation score to 1.42 ± 0.12 (*p* < 0.001 vs. COPD) (Figure 1A,B). ELISA analysis of BALF demonstrated that CS exposure elevated IL‐6 (COPD: 436.15 ± 38.95 pg/mL vs. sham: 10.23 ± 1.08 pg/mL, *p* < 0.001), TNF‐α (COPD: 423.61 ± 38.95 pg/mL vs. sham: 45.36 ± 4.15 pg/mL, *p* < 0.001), and IL‐1β (COPD: 218.63 ± 18.75 pg/mL vs. sham: 11.26 ± 1.05 pg/mL, *p* < 0.001), which were significantly attenuated by OMT treatment (IL‐6: 118.31 ± 9.63 pg/mL; TNF‐α: 186.91 ± 16.33 pg/mL; IL‐1β: 85.91 ± 7.89 pg/mL; all *p* < 0.001 vs. COPD) (Figure 1C–E). Pulmonary function tests revealed COPD mice showed impaired lung function, including elevated functional residual capacity (COPD: 0.58 ± 0.06 mL vs. sham: 0.33 ± 0.03 mL, *p* < 0.001), total lung capacity (COPD: 2.12 ± 0.21 mL vs. sham: 1.43 ± 0.14 mL, *p* < 0.01), chord compliance (COPD: 0.11 ± 0.01 mL/cmH_2_O vs. sham: 0.06 ± 0.008 mL/cmH_2_O, *p* < 0.001), forced vital capacity (COPD: 1.75 ± 0.17 mL vs. sham: 1.22 ± 0.12 mL, *p* < 0.01), and lung resistance (COPD: 1.26 ± 0.13 cmH_2_O·s/mL vs. sham: 0.77 ± 0.07 cmH_2_O·s/mL, *p* < 0.001), along with reduced FEV50/FVC ratio (COPD: 0.56 ± 0.06 vs. sham: 0.91 ± 0.09, *p* < 0.01). OMT treatment effectively normalized these parameters (functional residual capacity: 0.38 ± 0.04 mL; total lung capacity: 1.53 ± 0.15 mL; chord compliance: 0.075 ± 0.008 mL/cmH_2_O; forced vital capacity: 1.36 ± 0.14 mL; lung resistance: 0.85 ± 0.08 cmH_2_O·s/mL; FEV50/FVC: 0.84 ± 0.09; *p* < 0.05 or *p* < 0.01 vs. COPD) (Figure 1F–K), demonstrating its therapeutic efficacy in improving pulmonary mechanics.

### 3.2 OMT Treatment Ameliorates CS‐Induced Pulmonary Fibrosis and EMT in COPD Mice

**FIGURE 2:**
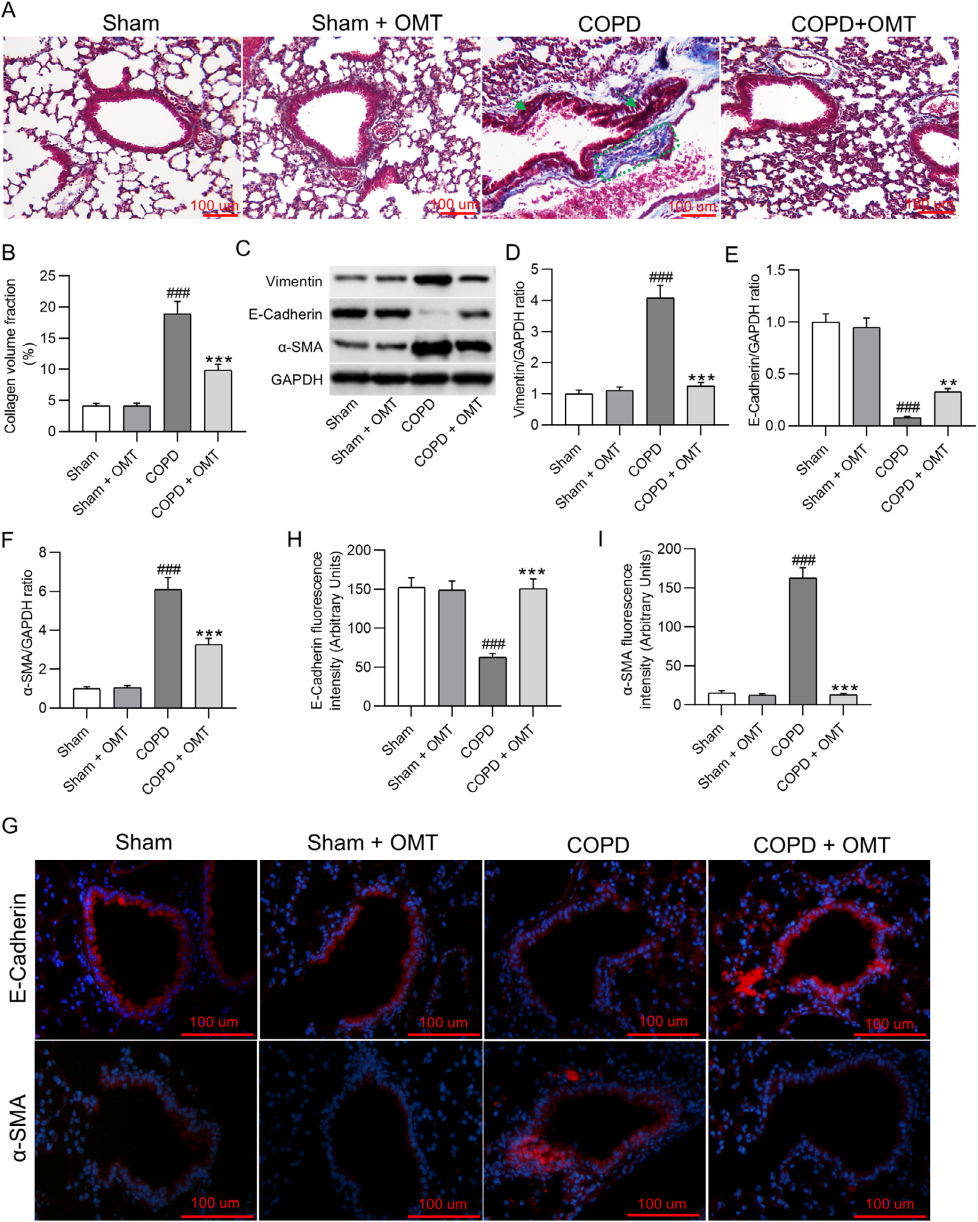
OMT treatment ameliorates CS‐induced pulmonary fibrosis and EMT in COPD mice. (A, B) Representative images of Masson‐stained lung sections (scale bar: 100 μm) with quantitative analysis of collagen deposition. Green dashed lines outline areas of collagen accumulation, and green arrows indicate airway wall thickening caused by extracellular matrix remodeling. Collagen deposition is a percentage of the total tissue area (mean ± SD). (C–F) Western blot analysis of EMT‐associated proteins (Vimentin, E‐Cadherin, α‐SMA) in lung tissue samples. Protein expression levels were normalized to GAPDH and are shown as fold changes relative to sham controls (mean ± SD). (G) Immunofluorescence staining of E‐cadherin and α‐SMA in lung tissues (scale bar: 100 μm). (H–I) Quantification of E‐cadherin intensity and α‐SMA intensity (RFU). *N* = 8 mice group. ^###^ *p* < 0.001 versus sham; **p < 0.01, ***p < 0.001 versus COPD.

Masson staining revealed significant collagen deposition in COPD mice (18.96% ± 1.96%) compared to sham controls (4.11% ± 0.42%, *p* < 0.001), which was significantly attenuated by OMT treatment (9.85% ± 0.97%, *p* < 0.001; Figure 2A,B). Western blot analysis showed that CS exposure significantly increased expression of Vimentin (4.08 ± 0.41‐fold vs. sham: 1.00 ± 0.12, *p* < 0.001) and α‐SMA (6.11 ± 0.61‐fold vs. sham: 1.00 ± 0.10, *p* < 0.001), while suppressing E‐Cadherin levels (0.08 ± 0.01‐fold vs. sham: 1.00 ± 0.08, *p* < 0.001). OMT administration reversed these alterations, restoring Vimentin (1.25 ± 0.12‐fold, *p* < 0.001 vs. COPD), α‐SMA (3.26 ± 0.33‐fold, *p* < 0.001 vs. COPD), and E‐Cadherin expression (0.33 ± 0.03‐fold, *p* < 0.01 vs. COPD) (Figure 2C–F). Immunofluorescence analysis demonstrated a decrease in E‐Cadherin intensity (from 152.31 ± 12.58 RFU in sham to 62.47 ± 5.32 RFU in COPD, *p* < 0.001) and an increase in α‐SMA intensity (from 12.52 ± 3.01 to 162.60 ± 13.44, *p* < 0.001), which were normalized to 150.78 ± 12.54 RFU (*p* < 0.001) and 12.96 ± 1.98 RFU (*p* < 0.001), respectively, following OMT intervention (Figure 2G–I).

### 3.3 Safety and Toxicity Evaluation of OMT

**FIGURE 3:**
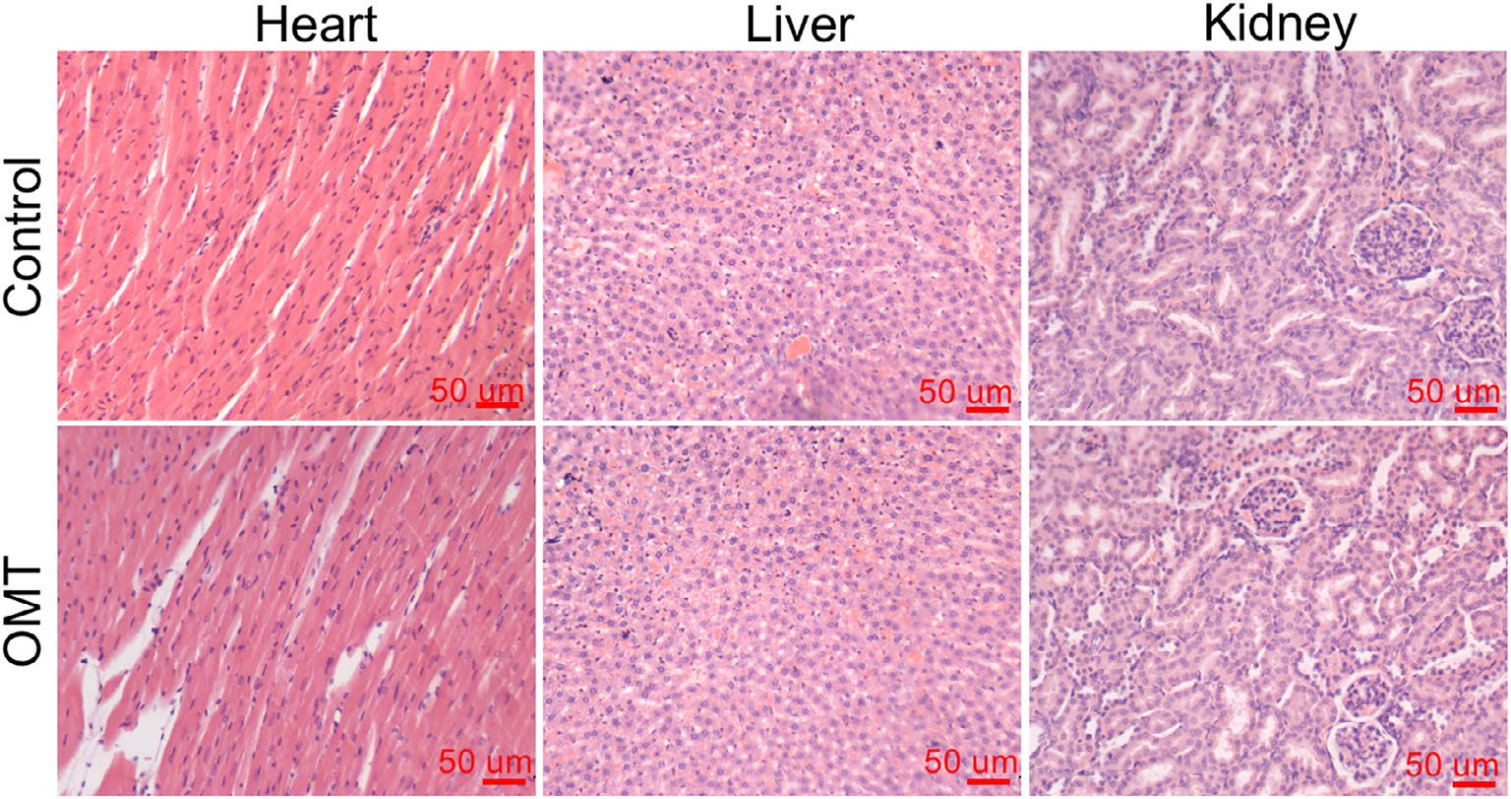
Safety and toxicity evaluation of OMT. H&E staining of heart, liver, and kidney tissues from sham and OMT‐treated mice (50 mg/kg, intraperitoneal injection daily for 3 months) (scale bar: 50 μm). *N* = 8 mice group.

H&E staining was conducted on heart, liver, and kidney tissues from both control and OMT‐treated groups to assess the systemic safety profile of OMT. Following OMT treatment, histological evaluation revealed no evident pathological alterations such as necrosis, inflammation, or fibrosis in any of the examined organs (Figure 3). These findings indicate that OMT administration at 50 mg/kg does not elicit observable off‐target toxicity.

### 3.4 OMT Treatment Protects HBE Cells From CSE or TGF‐β1‐Induced EMT

**FIGURE 4:**
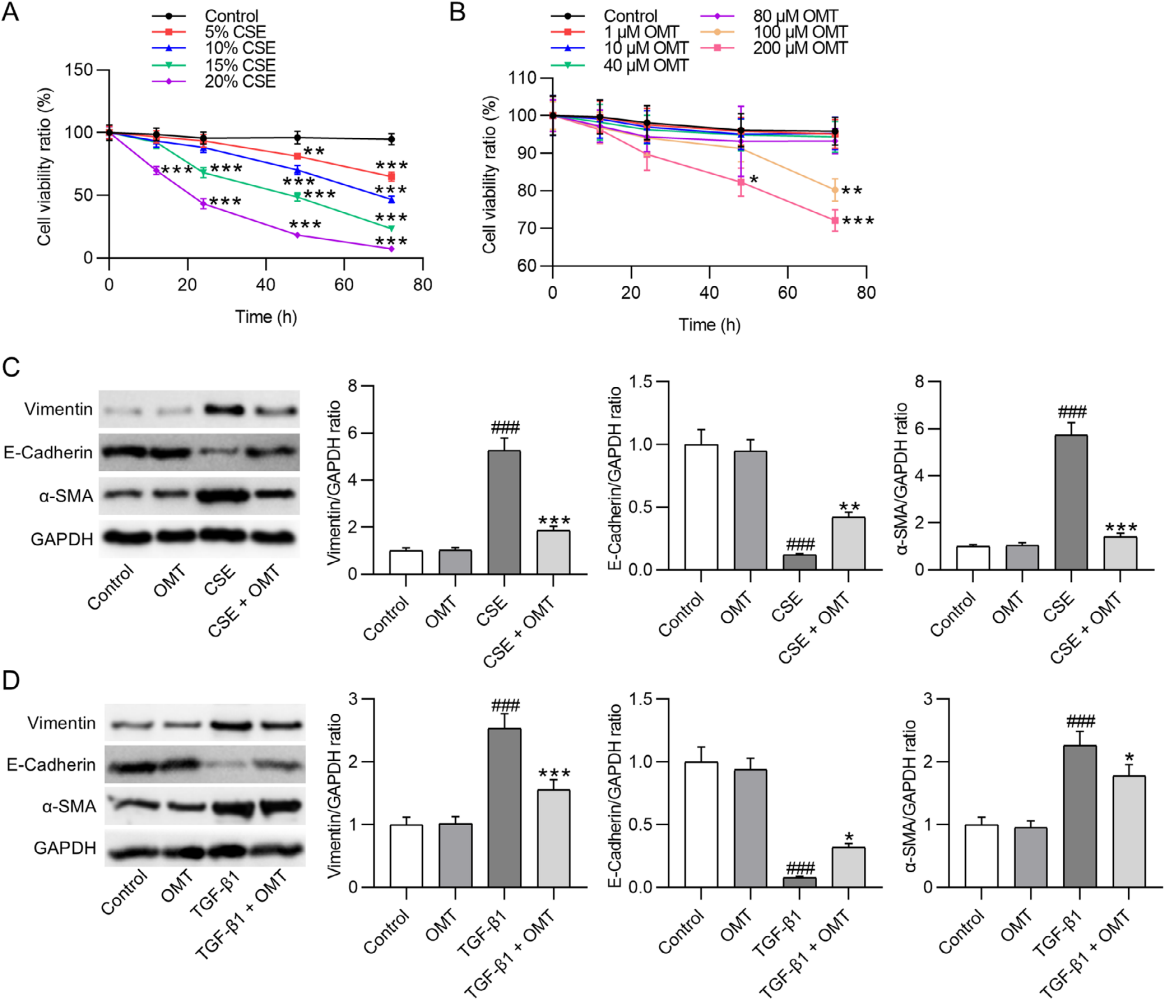
OMT attenuates CSE‐ and TGF‐β1‐induced EMT in HBE cells. (A) Cell viability was measured by CCK‐8 assay following exposure to 5%, 10%, 15%, or 20% (v/v) CSE for 12, 24, 48, or 72 h. (B) Cytotoxic effects of OMT at concentrations of 1, 10, 40, 80, 100, and 200 μM on HBE cells assessed over 12–72 h. Viability is a percentage relative to control (mean ± SD). (C, D) Western blot analysis of EMT‐related proteins (Vimentin, E‐Cadherin, α‐SMA) in HBE cells treated with 10% CSE (v/v, 48 h) or 10 ng/mL TGF‐β1 (48 h), with or without 24‐h pretreatment using 80 μM OMT. Protein levels normalized to GAPDH and expressed as fold change vs. control (mean ± SD). *N* = 3. ^###^ *p* < 0.001 versus control; *p < 0.05, **p < 0.01, ***p < 0.001 versus CSE/TGF‐β1.

For the in vitro experiments, HBE cells were cultured with 5%, 10%, 15%, or 20% (v/v) CSE for 12, 24, 48, or 72 h, and cell viability was quantified using the CCK‐8 assay. CSE reduced HBE cell viability in a dose‐ and time‐dependent manner, with 10% (v/v) CSE treatment for 48 h yielding a viability reduction of < 50% (Figure 4A). Cytotoxicity screening revealed that exposure to 100 μM or 200 μM OMT for 72 h significantly reduced HBE cell viability to 80.25% ± 2.96% and 72.11% ± 2.85%, respectively, while concentrations ≤ 80 μM revealed no cytotoxic effects (Figure 4B). Accordingly, 80 μM was chosen for subsequent experiments. Western blot analysis showed that stimulation with 10% CSE (48 h) significantly increased the expression of mesenchymal markers Vimentin (5.26 ± 0.53‐fold) and α‐SMA (5.75 ± 0.52‐fold) and decreased the epithelial marker E‐Cadherin (0.12 ± 0.01‐fold) in HBE cells. These alterations were significantly reversed by OMT pretreatment (80 μM) (*p* < 0.01 or *p* < 0.001; Figure 4C). A similar trend was observed in cells exposed to TGF‐β1 (10 ng/mL), where OMT attenuated the upregulation of Vimentin (3.5 ± 0.2‐fold) and α‐SMA (2.26 ± 0.23‐fold), as well as the suppression of E‐Cadherin (0.08 ± 0.01‐fold) (*p* < 0.05 or *p* < 0.001; Figure 4D).

### 3.5 OMT Treatment Inhibits the TGF‐β1/Smad Signaling Pathway

**FIGURE 5:**
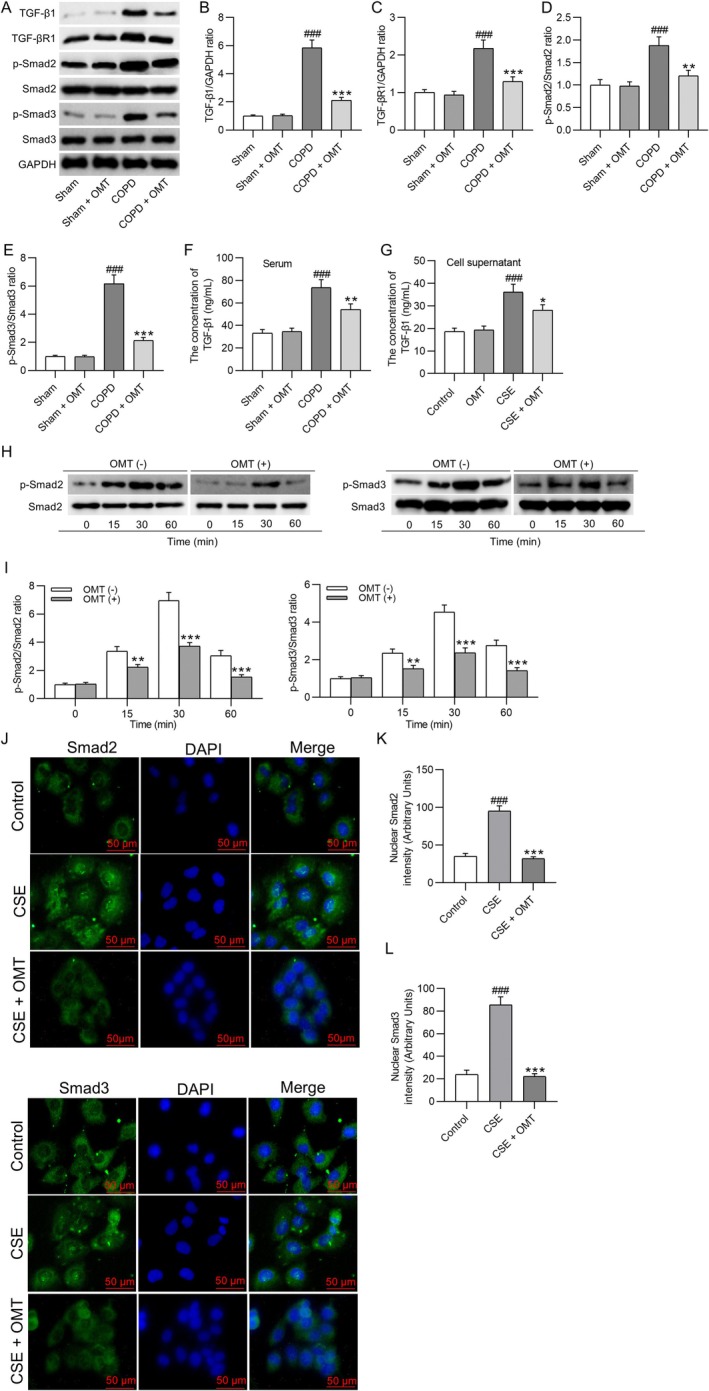
OMT treatment inhibits the TGF‐β1/Smad signaling pathway. (A–E) Western blot analysis of TGF‐β1, TGF‐βR1, p‐Smad2, Smad2, p‐Smad3, and Smad3 protein levels (fold change normalized to GAPDH or total Smad2/3) in lung tissues from sham, COPD, and COPD + OMT mice. (F) Serum TGF‐β1 concentration (ng/mL) quantified by ELISA. *N* = 8 mice/group. (G) TGF‐β1 secretion (ng/mL) in HBE cell supernatants after 10% CSE (v/v, 48 h) ± OMT (80 μM, 24‐h pretreatment). (H–I) Time‐dependent phosphorylation of Smad2/3 (fold change normalized to total Smad2/3) in HBE cells exposed to 10% CSE (v/v, 0–60 min) ± OMT (80 μM, 24‐h pretreatment). (J–L) Immunofluorescence staining (scale bar: 50 μm) and quantification of nuclear Smad2/3 intensity (RFU) in HBE cells treated with 10% CSE (v/v, 30 min) ± OMT (80 μM, 24‐h pretreatment). *N* = 3. ^###^ *p* < 0.001 versus sham/control; *p < 0.05, **p < 0.01, ***p < 0.001 versus COPD/CSE.

Western blot analysis demonstrated that lung tissues from CS‐exposed COPD mice showed significantly elevated levels of TGF‐β1 (5.86 ± 0.54‐fold), TGF‐βR1 (2.18 ± 0.22‐fold), phosphorylated Smad2 (p‐Smad2, 1.88 ± 0.19‐fold), and phosphorylated Smad3 (p‐Smad3, 6.18 ± 0.62‐fold) compared to sham controls (*p* < 0.001). Administration of OMT (50 mg/kg) significantly reduced these protein levels by 64%, 41%, 36%, and 65%, respectively (*p* < 0.01 or *p* < 0.001 vs. COPD; Figure 5A–E). ELISA results showed that serum TGF‐β1 levels increased from 33.28 ± 3.16 ng/mL (sham) to 73.69 ± 7.08 ng/mL in COPD mice (*p* < 0.001), whereas OMT treatment significantly lowered this value to 54.37 ± 4.85 ng/mL (*p* < 0.01; Figure 5F). In vitro, stimulation of HBE cells with 10% CSE (v/v) raised TGF‐β1 secretion from 18.63 ± 1.52 ng/mL (control) to 36.11 ± 3.57 ng/mL (*p* < 0.001), which was attenuated to 28.06 ± 2.47 ng/mL following OMT pretreatment (80 μM, *p* < 0.05; Figure 5G). Time‐course Western blot analysis showed that CSE (10% v/v) induced maximal phosphorylation of Smad2 (6.97 ± 0.57‐fold increase) and Smad3 (4.55 ± 0.37‐fold increase) at 30 min (*p* < 0.001 vs. 0 min). In comparison, OMT pretreatment reduced p‐Smad2 and p‐Smad3 levels by 47% and 48%, respectively (*p* < 0.001, Figure 5H,I). Immunofluorescence quantification confirmed that nuclear‐localized Smad2/3 intensity decreased from 95.33 ± 6.89 and 85.67 ± 7.12 in CSE‐treated HBE cells to 32.15 ± 2.46 and 22.14 ± 2.50 after OMT treatment, respectively (*p* < 0.001; Figure 5J–L).

### 3.6 Combined OMT and TGF‐βR1 Inhibition Synergistically Attenuate CSE‐Induced EMT in HBE Cells

**FIGURE 6:**
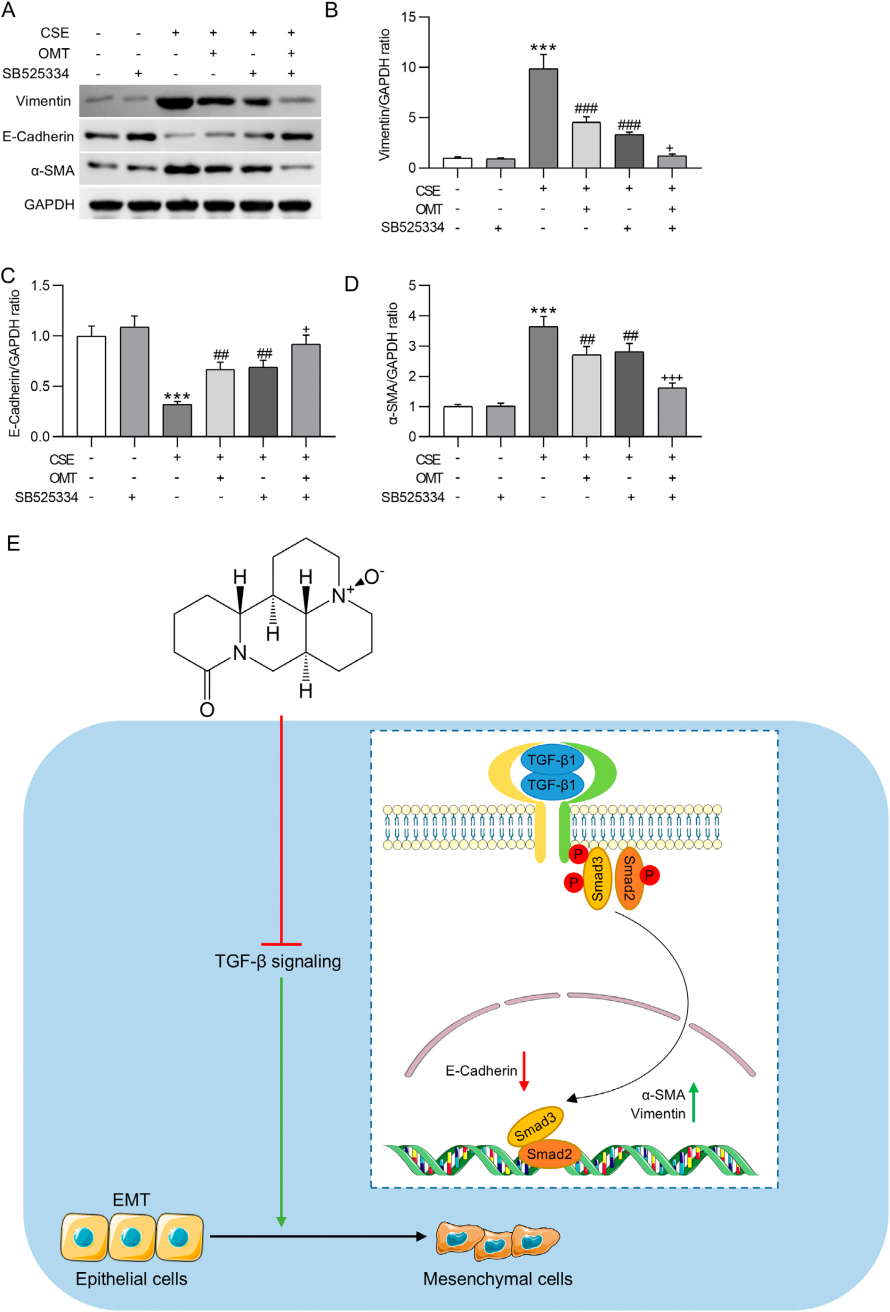
Treatment with SB525334 abolishes the suppression of OMT on CSE‐induced EMT in HBE cells. (A–D) Western blot analysis of EMT‐associated proteins (Vimentin, E‐Cadherin, α‐SMA) in HBE cells treated with 10% CSE (v/v, 48 h), 80 μM OMT (24‐h pretreatment), 10 μM SB525334 (TGF‐βR1 inhibitor, 48‐h pretreatment), or their combination. Protein levels normalized to GAPDH and expressed as fold change versus control (mean ± SD). (E) Schematic diagram illustrating the proposed mechanism of OMT in mitigating CS‐induced airway remodeling in COPD. *N* = 3. ***p < 0.001 versus control; ^##^ *p* < 0.01, ^###^ *p* < 0.001 versus CSE; ^+^ *p* < 0.05, ^+++^ *p* < 0.001 versus CSE + OMT/CSE + SB525334.

To confirm the involvement of the TGF‐β1/Smad signaling pathway in OMT‐mediated inhibition of EMT, CSE‐stimulated HBE cells were treated with the TGF‐βR1 inhibitor SB525334 (10 μM). SB525334 alone did not significantly alter EMT‐related protein expression in untreated cells. However, OMT and SB525334 individually reversed the CSE‐induced upregulation of mesenchymal markers and downregulation of epithelial markers. Combined treatment with OMT and SB525334 produced a synergistic effect, further reducing Vimentin (1.22 ± 0.16‐fold, *p* < 0.05) and α‐SMA (1.63 ± 0.15‐fold, *p* < 0.001), while restoring E‐Cadherin expression to near‐control levels (0.92 ± 0.09‐fold, *p* < 0.05) (Figure 6A–D).

## 4 Discussion

Our findings indicate that OMT attenuates CE‐induced airway remodeling in COPD by inhibiting EMT via suppressing the TGF‐β1/Smad signaling pathway. Unlike current clinical treatments such as bronchodilators (e.g., tiotropium) and anti‐inflammatory agents (e.g., fluticasone), which primarily target symptoms without halting fibrotic progression, OMT offers a dual‐action approach by simultaneously modulating EMT and inflammation, highlighting its potential as a disease‐modifying therapeutic candidate [28, 29]. Through integrated in vivo and in vitro models, OMT was identified as an effective regulator of EMT‐associated fibrosis and inflammation, offering mechanistic evidence to support its therapeutic potential.

Emerging evidence positions EMT as a critical mediator of airway remodeling, where epithelial cells acquire mesenchymal phenotypes through molecular reprogramming events [30]. In this study, OMT‐mediated restoration of EMT marker equilibrium was associated with improved pulmonary function parameters and decreased collagen deposition, supporting EMT modulation as a promising therapeutic approach for COPD. The TGF‐β1/Smad signaling axis was identified as a key regulatory mechanism, with persistent activation observed in COPD models evidenced by elevated TGF‐β1 levels, increased Smad2/3 phosphorylation, and nuclear accumulation of phosphorylated Smads. These results align with previous reports highlighting the central role of TGF‐β1 in CS‐induced pulmonary fibrosis and EMT progression [11, 31]. The observed suppression of TGF‐β1/Smad signaling by OMT offers mechanistic insight into its anti‐EMT activity. Although OMT's anti‐fibrotic effects through TGF‐β1/Smad inhibition have been reported in bleomycin‐induced pulmonary fibrosis [20, 23], this pathway had not been investigated in the context of COPD, where EMT is distinctly driven by chronic CS exposure and a persistently inflammatory microenvironment [4]. Importantly, our findings show that OMT‐mediated inhibition of EMT translates into measurable improvements in airflow obstruction (FEV50/FVC) and lung mechanics, effectively linking molecular modulation to functional outcomes. Moreover, the synergistic effect observed with combined OMT and TGF‐βR1 inhibitor SB525334 treatment marks a significant advancement. Previous studies evaluating OMT monotherapy in non‐COPD models [32, 33] did not investigate the benefits of dual targeting within the TGF‐β signaling axis. Our results indicate that co‐inhibition amplifies EMT suppression, highlighting OMT's potential to improve the efficacy of existing TGF‐β‐directed therapies and providing a strategic edge for COPD management.

Our study clarifies OMT's dual therapeutic action in COPD pathogenesis, integrating anti‐inflammatory and anti‐fibrotic effects. OMT significantly decreased BALF concentrations of IL‐6, TNF‐α, and IL‐1β while simultaneously reversing EMT‐associated collagen accumulation, demonstrating a multifaceted profile that sets it apart from existing single‐target treatments such as pirfenidone. Although pirfenidone, an approved idiopathic pulmonary fibrosis treatment, similarly modulates TGF‐β1 signaling [34], its limited COPD efficacy and frequent gastrointestinal toxicity [35] highlight OMT's therapeutic advantages. OMT demonstrated similar pathway inhibition at therapeutic doses without causing observable organ toxicity. Its distinctive ability to concurrently target both inflammation and fibrosis makes it a promising candidate for addressing the complex pathology of COPD. This dual‐action approach and a strong safety profile indicate that OMT may overcome the clinical shortcomings of current treatments that focus on only one aspect of the disease.

Although our results highlight OMT's therapeutic potential in CS‐induced COPD models, several limitations should be noted. Focusing solely on CS exposure, while mechanistically insightful, does not fully capture the clinical heterogeneity of COPD, where factors such as biomass smoke and α1‐antitrypsin deficiency also play significant roles. Although modulation of the TGF‐β1/Smad pathway is involved, it cannot be excluded that the concurrent involvement of other signaling pathways, such as IL‐6/STAT3 or Wnt/β‐catenin, is known to contribute to EMT progression in human airway epithelium, especially given the partial EMT reversal seen with OMT monotherapy. Furthermore, the specific molecular targets through which OMT influences TGF‐β1/Smad signaling remain to be identified. Future investigations using TGF‐β1 knockout models and phosphoproteomic analyses of OMT‐treated epithelial cells will be crucial to distinguish primary from secondary effects on these pathways.

In summary, this study identifies OMT as a promising therapeutic candidate for COPD by simultaneously modulating inflammatory responses and TGF‐β1/Smad‐driven EMT pathways (Figure 6E). Its capacity to reduce airway remodeling and a favorable safety profile highlight OMT's potential as a disease‐modifying treatment. These results offer strong preclinical support for advancing OMT into clinical trials for COPD management.

## Ethics Statement

All mice were treated humanely to alleviate their suffering following a protocol approved by the Institutional Animal Care and Use Committee of Xiangyang Traditional Chinese Medicine Hospital.

## Conflicts of Interest

The authors declare no conflicts of interest.

## Data Availability

The data that support the findings of this study are available from the corresponding author upon reasonable request.
